# Contribution of prostanoid FP receptor and prostaglandins in transient inflammatory ocular hypertension

**DOI:** 10.1038/s41598-018-29273-1

**Published:** 2018-07-23

**Authors:** Reiko Yamagishi-Kimura, Megumi Honjo, Makoto Aihara

**Affiliations:** Department of Ophthalmology, The University of Tokyo School of Medicine, Tokyo, Japan

## Abstract

We explored the involvement of FP receptor and endogenous prostaglandins (PGs) in transient ocular hypertension (OH) induced by PGE2 or PGF2α in mouse eyes. PGE2 and PGF2α were topically applied to induce transient OH in Wild-type (WT) and FP-, EP1-, EP2-, and EP3-deficient (knockout [KO]) mice. To suppress endogenous PG production, the non-steroidal anti-inflammatory drug nepafenac was applied topically before treatment. PGE2 and PGF2α induced significant OH in the WT, FPKO, and EP1–3KO mice compared to the control 30 min after instillation, and the increase in IOP at 30 or 60 min after instillation in FPKO mice was significantly higher than that in the WT mice. The effects of PGF2α on the increase in IOP were significantly weaker than those of PGE2, especially in EP1KO and EP3KO mice. Transient OH induced by PGE2 and PGF2α was significantly attenuated by nepafenac treatment in FPKO mice. Transient OH was induced by PGE2 and PGF2α in WT, FPKO, and EP1–3KO mice, which was enhanced in FPKO mice. This OH was significantly diminished by nepafenac treatment in FPKO mice, suggesting that FP receptor may have an important naïve physiological role in the eye, and could regulate IOP elevation during PG-associated ocular inflammation.

## Introduction

It has long been reported that inflammation-inducing substances, referred to as ‘irins’, are present in the eye, most of which have been clarified to be the prostaglandins (PGs) PGE2 and PGF2α^1^. PGs have been recognized as predominantly inflammation-inducing substances or pro-inflammatory molecules in the eye. However, drugs and analogs related to PGs are currently the most commonly used agents to treat glaucoma and lower intraocular pressure (IOP), although their physiological roles in the eye have not been fully clarified.

Current PG-related drugs have high selectivity for the FP receptor. PGE2 has four distinct receptors, EP1, EP2, EP3, and EP4, of which PGF2α binds to the FP receptor. Studies of drug receptor affinity and receptor-deficient (knockout [KO]) mice have reported that the FP receptor has a crucial role in reducing IOP^2–5^. In addition, the EP3, EP2, and EP4 receptors are involved in lowering IOP^6^. Involvement of both the FP receptor and endogenous PGs has been reported not only in reductions of IOP, but also in maintenance of IOP homeostasis^2,7–11^.

However, the physiological role of the FP receptor in the eye has not been clarified, having thus far been evaluated only under conditions of reduced IOP using externally applied FP agonists. In our previous study, we speculated that IOP might differ between FPKO and wild-type (WT) mice; however, we found that the baseline IOPs during the day and at night did not differ significantly between FPKO and WT mice^4^. Therefore, we speculated that the FP receptor is unlikely to be involved in the maintenance of IOP under normal conditions, but rather may have an endogenous role in controlling IOP fluctuations in the case of ocular inflammation. In ocular inflammation, PGE2 and PGF2α levels increase in the anterior chamber^1^. Clinically, fluctuations in IOP are often observed in uveitis. PGE2 and PGF2α not only lower IOP, but have also been reported to increase IOP transiently in the early phase after instillation in mice^6^, monkeys^12–14^, rabbits^15^, and humans^16^.

In this study, we investigated the involvement of the FP receptor and endogenous PGs in transient ocular hypertension (OH) induced by PGE2 or PGF2α in mouse eyes.

## Results

### Time-dependent IOP changes after treatment with PGE2 methyl ester in WT mice

**Figure 1:**
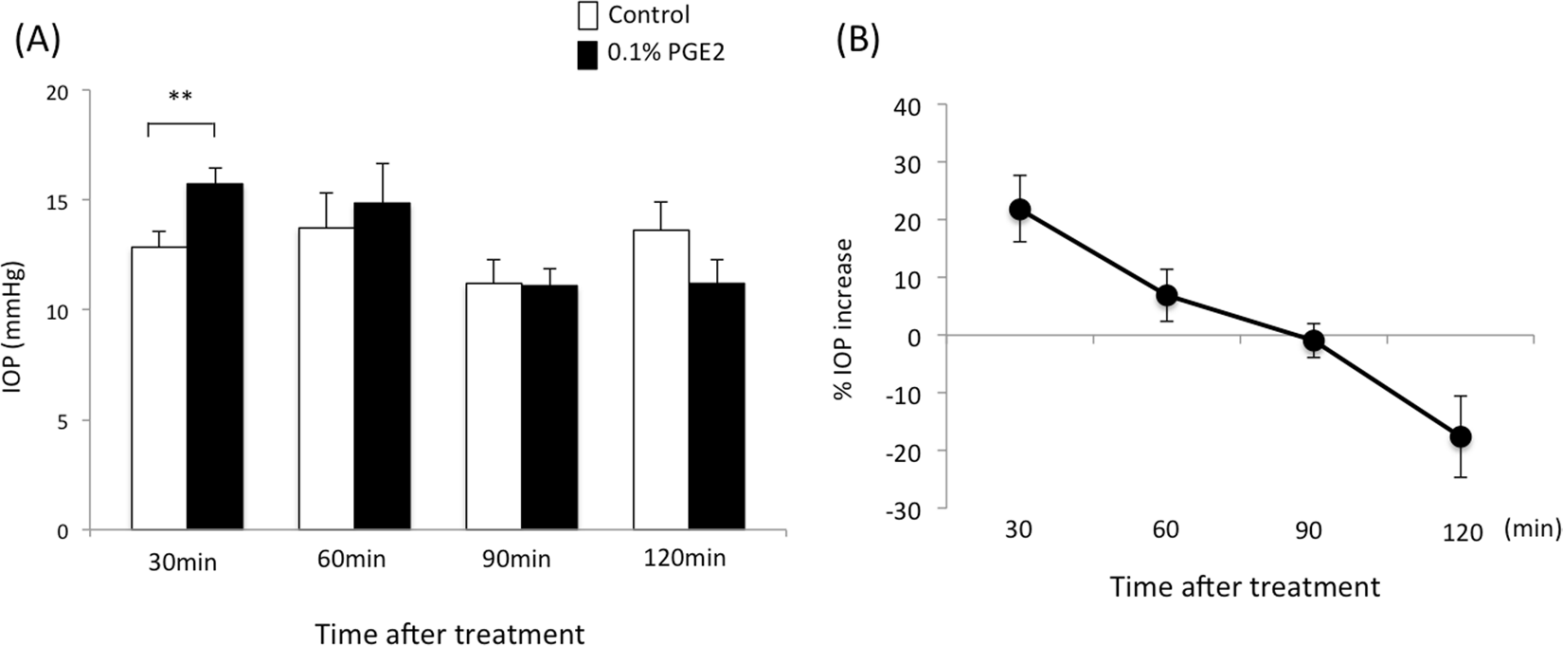
Time course of (**A**) intraocular pressure (IOP) change (mmHg) and (**B**) the IOP increase (%) after treatment with 0.1% PGE2 in wild-type (WT) mice. Data are expressed as means ± standard deviation (SD) (n = 5–10). **p < 0.01 for 0.1% PGE2-treated versus contralateral vehicle-treated eyes (control).

For preliminary experiments, IOP was measured in WT mice using Tonolab at 0, 30, 60, 90 and 120 min after topical instillation of 0.01% or 0.1% PGE2 and PGF2α. There were significant differences by PGE2 and PGF2α in lower concentration at 30 min after dosing compared to non-treated group. However, the IOP increase by 0.01% PGE2 or PGF2α was minor compare to 0.1% of both drugs (Supplement Figure S1) Therefore, we determined the dose for PGE2 and PGF2α as 0.1%. Next, we measured IOP in WT mice 30, 60, 90 and 120 min after topical instillation of 0.1% PGE2 or PGF2α (n = 5–10 at each time point). The IOP of each drug-treated group was compared to the vehicle control group using Student’s t-test. The IOPs at 30 min after instillation of the vehicle or 0.1% PGE2 were 12.8 ± 0.7 and 15.7 ± 0.7 mmHg, respectively, indicating that PGE2 significantly increased IOP compared to the control 30 min after instillation (p < 0.001) in WT mice (Fig. 1). The IOPs at 60, 90 and 120 min after instillation of the vehicle and 0.1% PGE2 were 13.7 ± 1.6 and 14.8 ± 1.8 mmHg at 60 min, 11.2 ± 1.1 and 11.1 ± 0.8 mmHg at 90 min, and 13.6 ± 1.3 and 11.2 ± 1.1 mmHg at 120 min, respectively. At 120 min after dosing, 0.1% PGE2 lowered IOP, significantly (p < 0.05). The IOP increase (%) between the PGE2-treated group and vehicle-treated control at 30, 60, 90 and 120 min after instillation were 21.9 ± 5.7, 6.8 ± 4.5, −0.9 ± 3.0 and −17.6 ± 7.1%, respectively, indicating that the IOP increase decreased in a time-dependent manner.

**Figure 2:**
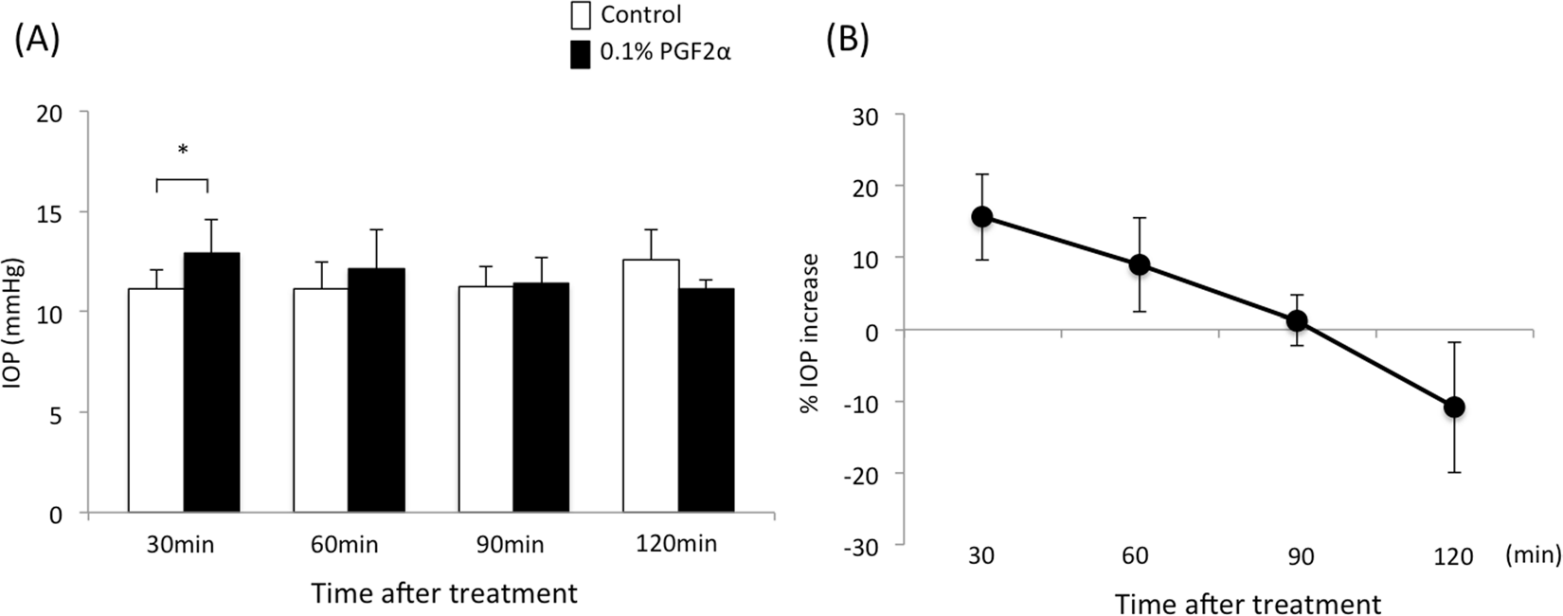
Time course of (**A**) IOP change (mmHg) and (**B**) the IOP increase (%) after treatment with 0.1% PGF2α in WT mice. Data are expressed as means ± SD (n = 5–9). *p < 0.05 for the treated versus contralateral vehicle-treated eyes (control).

Conversely, the IOPs at 30 min after instillation of the vehicle or 0.1% PGF2α were 11.2 ± 0.9 and 12.9 ± 1.7 mmHg, respectively, and 0.1% PGF2α significantly increased the IOP at 30 min after instillation in WT mice (p = 0.035) (Fig. 2). At 60, 90 and 120 min after instillation, the IOPs of vehicle-treated and PGF2α-treated eyes were 11.1 ± 1.3 and 12.2 ± 1.9 mmHg at 60 min, 11.2 ± 1.0, 11.4 ± 1.3 mmHg at 90 min, and 12.6 ± 1.5 and 11.1 ± 0.5 mmHg at 120 min; vehicle-treated and PGF2α-treated, respectively, and there were no significant differences in IOP between the PGF2α-treated group and vehicle-treated control. The IOP increase at 30, 60, 90 and 120 min after instillation were 15.6 ± 6.0, 8.9 ± 6.6, 1.2 ± 3.5 and −10.8 ± 9.1%, respectively after PGF2α treatment, and these decreased in a time-dependent manner. The effect of PGF2α on the IOP increase at 30 min after instillation was significantly weaker than that of PGE2 in WT mice (p = 0.037).

### Time-dependent IOP changes after 0.1% PGE2 methyl ester treatment in FPKO, EP1–3KO, and WT mice

**Figure 3:**
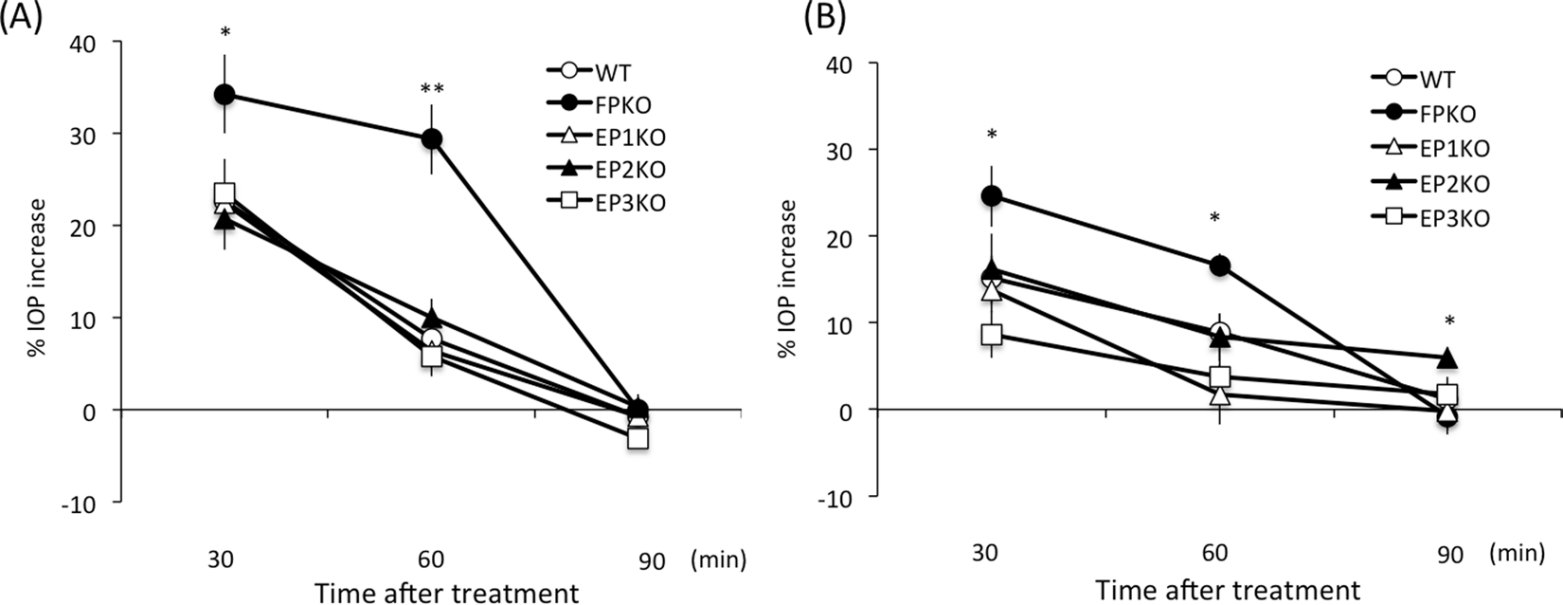
Time course of IOP increase with 0.1% (**A**) PGE2 and (**B**) PGF2α in WT, FPKO, EP1KO, EP2KO, and EP3KO mice. Data are expressed as means ± SD (n = 5–10). ***p < 0.05 or 0.01 for the WT versus KO mouse groups.

To explore the roles of the FP, EP1, EP2, and EP3 receptors in PGE2- and PGF2α- induced OH, we measured the IOP of WT, FPKO, and EP1–3KO mice at 30, 60, and 90 min after topical instillation of 0.1% PGE2 or PGF2α (n = 5–10 per time point). In preliminary experiment, there were no significant differences of IOP in all phenotype mice using microneedle methods (Supplementary Table S1). The IOP increase (%) 30 and 60 min after instillation of 0.1% PGE2 in WT, FPKO, and EP1–3KO mice were 22.7 ± 5.4, 34.2 ± 12.9, 22.3 ± 6.3, 20.7 ± 8.7, and 23.5 ± 10.9% and 7.7 ± 5.6, 29.3 ± 11.4, 6.4 ± 5.0, 10.0 ± 5.4, 5.8 ± 4.8%, respectively (Fig. 3). Treatment with 0.1% PGE2 significantly increased the IOP at 30 and 60 min after instillation in FPKO mice compared to the WT (p = 0.030 and 0.0003, respectively). At 90 min after PGE2 treatment, there were no differences in the IOP increase between the WT mice and KO mice.

Meanwhile, the IOP increase at 30 and 60 min after treatment with 0.1% PGF2α in WT, FPKO, and EP1–3KO mice were 15.1 ± 8.6, 24.6 ± 11.1, 13.8 ± 6.7, 16.2 ± 10.8, and 8.6 ± 6.7% and 8.9 ± 6.6, 16.6 ± 3.8, 1.7 ± 9.8, 8.4 ± 7.3, and 3.8 ± 8.0%, respectively. Moreover, 0.1% PGF2α significantly increased the IOP at 30 and 60 min after instillation in FPKO mice compared to the WT (p = 0.045 and 0.010, respectively). There were no differences in the IOP increase between the WT and the other types of mice at 90 min after instillation of PGF2α. However, 0.1% PGF2α significantly increased the IOP at 90 min after instillation in EP2KO mice compared to the WT (p = 0.016) (Fig. 3B).

Both PGE2 and PGF2α increased the IOP significantly in WT, FPKO, and EP1–3KO mice at 30 min after instillation; however, the influence of PGF2α on the increase in IOP was significantly weaker than that of PGE2, especially in EP1KO and EP3KO mice (p = 0.023 and 0.0061, respectively).

### Involvement of endogenous PG production in transient OH by PGE2 and PGF2α

**Figure 4:**
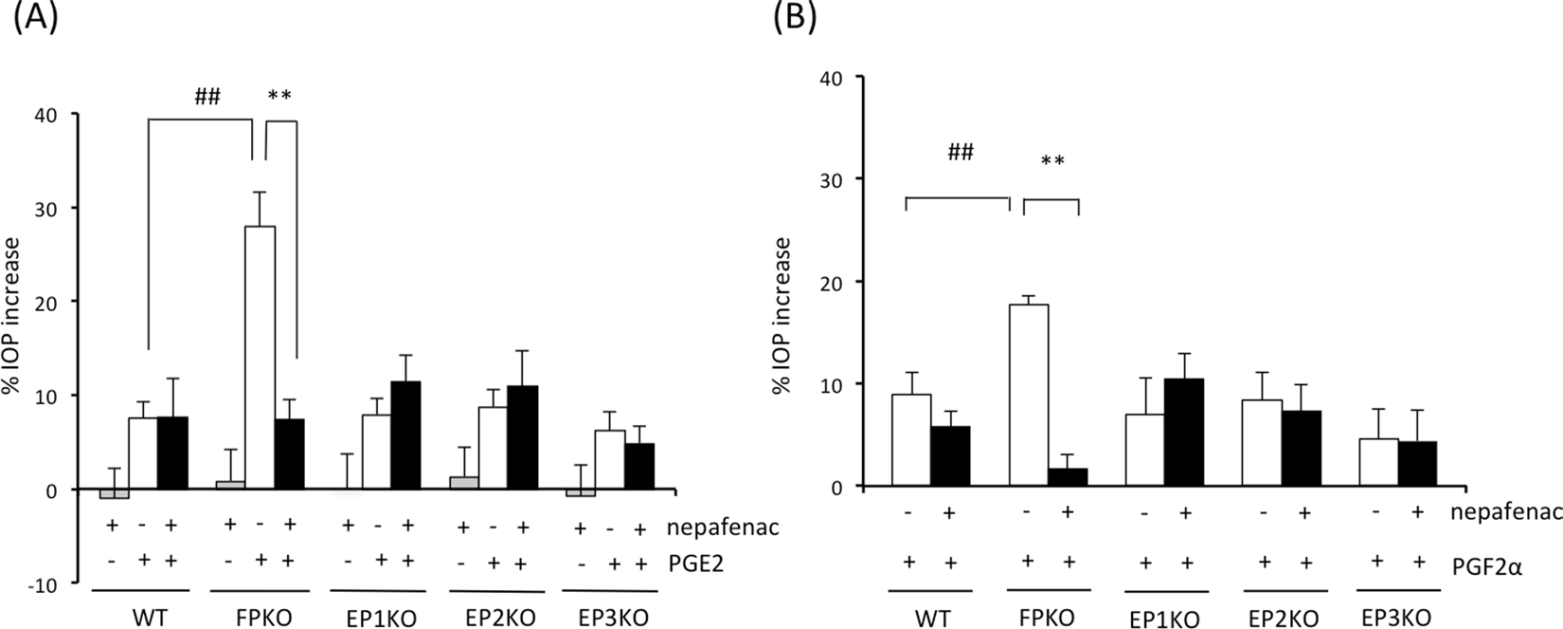
Influence of the nonsteroidal anti-inflammatory drug (NSAID) nepafenac on transient ocular hypertension (OH) induced by (**A**) PGE2 and (**B**) PGF2α in WT, FPKO, EP1KO, EP2KO, and EP3KO mice. Data are expressed as the means ± standard error of the mean (n = 6–8 per column). ^##^p < 0.01 for WT versus FPKO based on the Mann-Whitney U test. **p < 0.01 for NSAID (−) versus NSAID (+) based on the Mann-Whitney U test.

Transient OH may be derived from endogenously produced PGs. Therefore, we treated mouse eyes with the nonsteroidal anti-inflammatory drug (NSAID) nepafenac (0.1%). After nepafenac treatment, IOP was measured in WT, FPKO, and EP1–3KO mice 60 min after topical instillation of 0.1% PGE2 or PGF2α. As shown in Fig. 3, the IOP increase ratio was higher at 30 min after dosing than 60 min, but the difference of IOP increase between WT mice and FPKO mice was more significant at 60 min after dosing than 30 min. (p value of WT vs. FPKO was 0.03 and 0.0003. at 30 min and 60 min after dosing, respectively). Therefore, we have selected a time point of 60 min after dosing in order to elucidate the influence of endogenous PGs. Treatment with nepafenac-only showed no effects on IOP in all phenotypes of mice (gray columns, Fig. 4A). After PGE2 treatment, the IOP increase with and without nepafenac treatment in WT mice were 7.5 ± 5.3 and 7.6 ± 11.6%, respectively. No significant effect of nepafenac on PGE2-induced OH was observed in WT mice. There were no significant differences in the IOP increase between non-treatment and nepafenac treatment in EP1–3KO mice (Fig. 4A). Conversely, the IOP increase in non-treated and nepafenac-treated FPKO mice were 27.9 ± 11.6 and 7.4 ± 5.7%, respectively, and PGE2-induced OH in FPKO mice was significantly attenuated by nepafenac treatment (p = 0.00028) (Fig. 4A).

Likewise, under the 0.1% PGF2α treatment, the IOP increase with and without 0.1% nepafenac treatment in WT mice were 8.9 ± 6.6 and 5.8 ± 4.3%, respectively, which did not differ significantly. Moreover, there were no significant differences in the IOP increase between non-treatment and nepafenac treatment in EP1–3KO mice (Fig. 4B). However, the IOP increase in the non-treated and nepafenac-treated FP mice were 17.7 ± 2.4 and 1.7 ± 3.3%, respectively, and PGF2α-induced OH was significantly diminished by nepafenac treatment in FPKO mice (p = 4.6E-6) (Fig. 4A). The difference between WT and FPKO in presence of PGF2α and nepafenac was not statistically significant. (p = 0.0702).

## Discussion

We found that transient OH induced by PGE2 and PGF2α in WT mice was enhanced in FPKO mice. This suggests that the FP receptor may have an important physiological role in stabilizing IOP fluctuations in PG-associated OH. In general, the physiological role of the FP receptor has been implicated in uterine contractions and corpus luteum regression. Previously, there are many reports about IOP changes by PGE2 or PGF2α instillation, but there were no reports to measure IOP in earlier phase in mice eyes like as our experiment. Our results show the fine and rapid changes of IOP occurring very early phase using microneedle methods. Moreover, there are no reports of the endogenous function of the FP receptor in the eye. Our understanding of the FP receptor is limited, being based only on its response to externally administrated agents (i.e., PG analogues) in glaucoma treatment. Thus, this is the first report to clarify a ‘naïve’ role of the FP receptor in the eye, which may act as a regulator of IOP elevation in cases of PG-associated ocular inflammation.

Several physiological challenges are known to increase IOP, such as surgical invasion and inflammation; however, the precise mechanisms involved in OH are not well understood^17–25^. PGE2 is known to be released under such conditions. Similarly, in this study, topical instillation of PGE2 resulted in transient OH (Figs 1, 3, and 4); therefore, we speculate that PGE2 may be a major contributor to OH in ocular inflammation. However, our study suggested that the EP1, EP2, and EP3 receptors were not directly related to this mechanism, because there were no significant changes in IOP fluctuation in the respective KO mice (Fig. 3A). Previously, PGE2-induced transient OH was attenuated by dipyridamole, which inhibited cAMP-phosphodiesterase and cAMP production^26^. Thus, PGE2-induced transient OH may partially act via an increase in cAMP after the activation of the G protein-coupled receptors EP2 and EP4, although it is unknown whether this pathway is directly or indirectly activated through other molecules. Unfortunately, the role of EP4 was not clarified in our study, because EP4 deficiency is embryonically lethal in mice. Therefore, EP4 may have an important role in increased IOP. Future studies should include conditional KO, multiple KO mice, or antagonists for each receptor to clarify the underlying mechanism of OH.

Similar to PGE2, transient OH was observed after PGF2α administration in this study, but the response was weaker than that for PGE2. The current preferred glaucoma drugs, PG analogues, are PGF2α derivatives developed with the aim of increasing affinity for the FP receptor; however, PGF2α also has an affinity for EP receptors^27^. Therefore, we speculate that the IOP increase by PGE2 or PGF2α was mediated by nonspecific activity of PGE2 or PGF2α, considering a previous report that PGF2α affect not only FP receptors but also other prostanoid receptors^27^. In our previous mouse study, current PG analogues caused a significant IOP reduction without OH in the early phase^28^. Taken together, partial activation of EP receptors may contribute to the transient OH induced by PGF2α. Thus, our results indicate that the mechanisms of induction of transient OH are similar for both PGE2 and PGF2α, being mediated by EP receptors.

**Figure 5:**
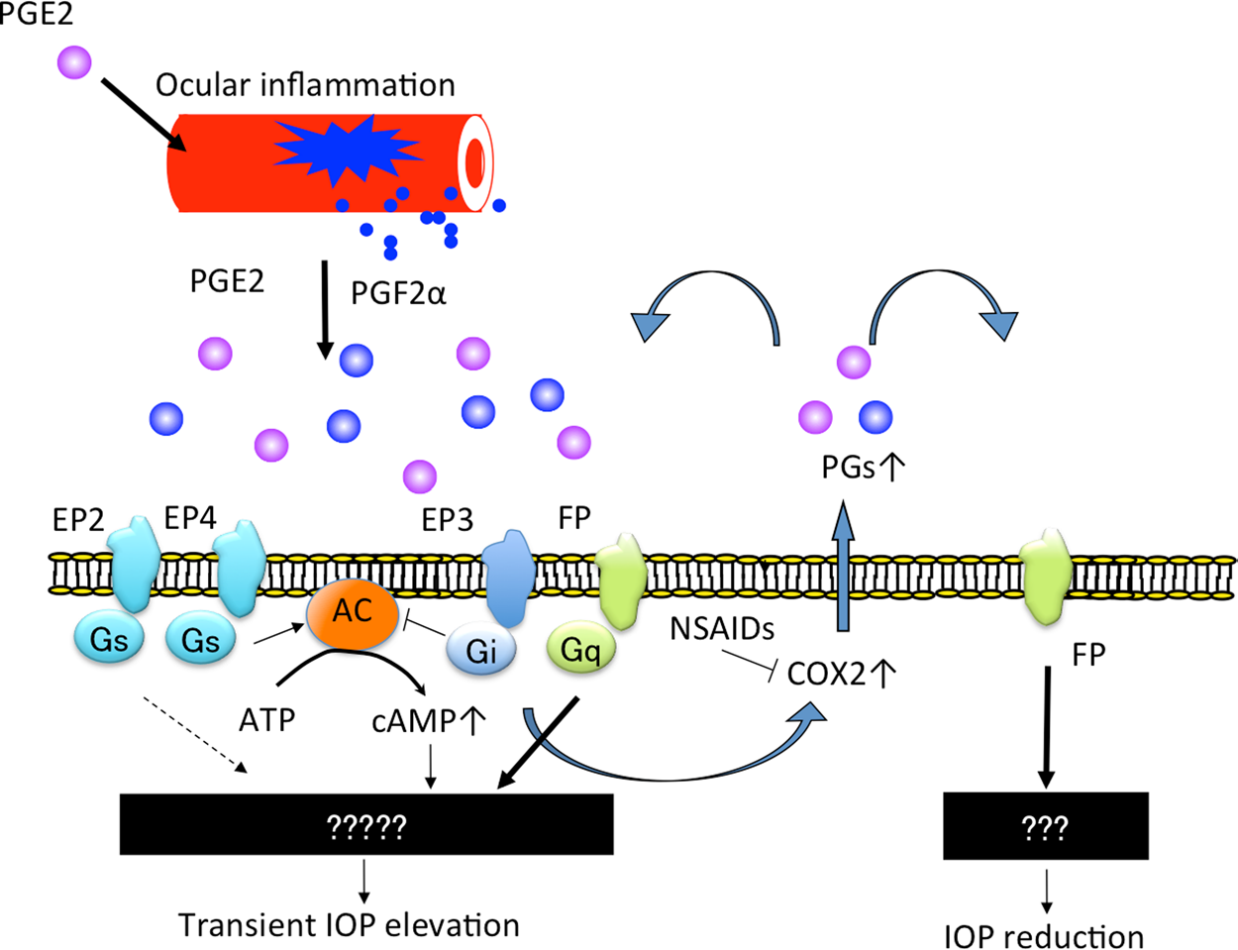
Involvement of PGE2, PGF2α and FP receptors in regulation of IOP.

The FP receptor has an important role in suppressing PGE2-induced transient OH. Moreover, transient OH induced by PGE2 and PGF2α was significantly attenuated by NSAID treatment in FPKO mice in this study, but not in the other KO or WT mice (Fig. 4). This indicated that endogenous PGF2α was produced after PGE2 or PGF2α stimulation. It has been previously reported that cyclooxygenase (COX) activity is induced by FP receptor stimulation^29^. These results suggest that, in ocular inflammation, the levels of various PGs, acting as local mediators, increase via COX activation and subsequently produced enzymes, and that PGE2 acts on EP receptors, ultimately leading to an increase in IOP via cAMP augmentation or other unknown mechanisms. Simultaneously, enhanced PGF2α also stimulates FP receptors, leading to a reduction in IOP. Further, it is possible that non-PG molecules (e.g. thromboxanes or other downstream molecules of the COX-2 pathway) involved in the IOP increase since the NSAID abrogates the IOP increase even in the presence of PG treatment. This actually indicates that PG treatment might be inducing other IOP elevating factors, however, it was not easy to prove this in the present study because there are various factors involved in the maintenance of IOP. However, at least in the present study, it was clarified that the cox-activity induced by the stimulation of FP receptors or other receptors was involved in homeostasis maintenance of IOP when IOP was elevated by certain stimuli. These mechanisms are summarized in a schematic diagram of our hypothesis (Fig. 5). If the FP receptor does not sufficiently respond to inflammation, the eye may be exposed to high pressure, inducing optic disc damage or decreased ocular blood flow. However, this speculation is based only on the effects of NSAID treatment and prostanoid receptor deficiencies on PG-induced OH, and further studies are needed to clarify the action of PGs in the aqueous fluid.

Several limitations of the present study must be addressed. First, we measured IOP invasively using the microneedle method; therefore, we could not measure or compare IOP, within the same eye, at baseline and each subsequent time point. Ideally, we should have measured IOP using a non-invasive device, such as the Icare® Tonolab (Icare Finland Oy, Vantaa, Finland), in a time-dependent manner after instillation. However, the accuracy of the Tonolab was too low to evaluate transient OH induced by PGE2 and PGF2α; therefore, we used the microneedle method. It is necessary to improve the accuracy of tonometric measurements of IOP in mice for future studies. Second, we used concentrations of PGE2 and PGF2α of 0.1%, based on the results of preliminary experiments in which elevated IOP was confirmed by topical instillation. However, the actual drug concentration in the eye was unknown, and it was unclear whether it clinically reflected the concentration of PGs that may occur in the eye during inflammation. In the present study, we could not measure the PG concentration in the eye using mass spectrometry due to the low volume of aqueous humor in mouse eyes. Therefore, future studies should aim to increase the sensitivity of such measurements and determine the PG concentration in the eye. Finally, our results have not completely elucidated the role of FP receptors in the present study, although we speculate that FP receptors may act to lower IOP when ocular hypertension is induced by certain stimulus. We may get more new knowledge when we could do further experiments using antagonists of these receptors. However, unfortunately at present, as far as we know, there are no suitable antagonists for these receptors having high selectivity, and there are no evidences that these antagonists act topically. We would like to consider these experiments using antagonist as an important task in the future.

In conclusion, we clarified that PGE2 and PGF2α induced transient OH in mouse eyes through secondary induction of endogenous PGs, and this transient OH was suppressed by FP receptor stimulation, a mechanism that is often exploited to lower IOP via external administration of PG analogues. Although elucidating the mechanism underlying IOP fluctuation adjustment and control in the eye, in the case of disease, inflammation, or even normal status, remains a challenge, we showed that the FP receptor might have a biological role in lowering or adjusting IOP against endogenous PG production *in vivo*. Elucidating the factors controlling the IOP could lead to the establishment of a new treatment for glaucoma.

## Materials and Methods

### Materials

We used PGE2 methyl ester (Cayman Chemical, Ann Arbor, MI, USA), PGF2α methyl ester, and nepafenac (Nevanac® ophthalmic solution 0.1%; donated by Alcon Pharma, Tokyo, Japan) as the NSAID solution in this study.

### Animals

All animals used in this study were treated in accordance with the Association for Research in Vision and Ophthalmology Statement for the Use of Animals in Ophthalmic and Vision Research, as well as the rules outlined by the local Animal Use Committee of the University of Tokyo. The Ethics Committee for Animal Experiments at University of Tokyo approved all experimental procedures. As WT mice, male C57BL/6 J mice were purchased from Japan Tokyo Laboratory Animals Science Co. Ltd. (Tokyo, Japan). FPKO, EP1KO, EP2KO, and EP3KO mice^30,31^ were donated by the Department of Drug Discovery Medicine, Kyoto University Graduate School of Medicine (Kyoto, Japan). Mice were bred and housed in clear cages loosely covered with air filters. The cages contained white chip bedding. The temperature was maintained at 21 °C under a 12:12-h light:dark cycle. All mice had access to food and water ad libitum. We used mice older than 8 weeks of age.

### Preparation and instillation of ophthalmic solutions

PGE2 methyl ester and PGF2α methyl ester were stored at −80 °C in 100% dimethyl sulfoxide (DMSO) and were diluted with phosphate-buffered saline immediately before use to yield a 5% DMSO concentration. We used the ester form of PGs as eye drops because this chemical structure readily penetrates into the anterior chamber under a low concentration of drug. With a micropipette, 3 µL of each drug solution was topically applied in a masked manner to one eye selected at random, and the other eye was used as a vehicle-treated control.

### Mouse IOP measurement

We used two instruments to measure IOP; the microneedle method and he rebound tonometer (TonoLab; Icare®, Vantaa, Finland). IOP was measured directly by the microneedle method in anesthetized mice as previously described^32^. Briefly, a microneedle made of borosilicate glass (75–100 μm in tip diameter, 1.0 mm in outer diameter; World Precision Instruments, Sarasota, FL, USA) was connected to a pressure transducer (model BLPR; World Precision Instruments). The pressure detected by the transducer was recorded with a data acquisition and analysis system (PowerLab; ADInstruments, Colorado Springs, CO, USA). The microneedle was placed in the anterior chamber, and the pressures in both eyes were recorded in mice under ketamine/xylazine anesthesia. With the TonoLab, automatically averaged readings were recorded. The IOP was measured in conscious mice. All measurements were performed from 21:00–23:00.

We randomly applied 3 µL of saline, 0.1% PGE2 methyl ester (PGE2), and 0.1% PGF2α methyl ester (PGF2α) to one eye of each mouse (n = 7–10 per group). The vehicle-treated contralateral eye served as a control. The effect of each drug was calculated as 100 × (IOP of treated eye – IOP of contralateral eye)/IOP of contralateral eye (%) in each mouse, referred to as the IOP increase. In another series of experiments, the NSAID nepafenac (Nevanac® ophthalmic solution 0.1%) was topically administered to each mouse in both eyes 30 min before the topical administration of PGE2 or PGF2α to suppress endogenous PG production, and the IOP was measured 1 h after administration of PGE2 or PGF2α.

### Data and statistical analysis

All results are presented as the means ± standard deviation (SD). Statistical analyses were performed using JMP Pro 11 software (SAS Institute Inc., Cary, NC, USA). Student’s *t*-test with the post-hoc Bonferroni test was used for between-group comparisons. Dunnett’s multiple comparison test was employed to compare more than two groups. All data represent the means of at least three independent experiments. Differences were considered to be statistically significant when p < 0.05.

## Electronic supplementary material

Supplementary tableS1, Figure S1
