# Supplementary material for: Contribution of prostanoid FP receptor and prostaglandins in transient inflammatory ocular hypertension
Source: Sci Rep. 2018 Jul 23;8:11098. doi: 10.1038/s41598-018-29273-1 (PMC6056481; doi:10.1038/s41598-018-29273-1)

**Contribution of prostanoid FP receptor and prostaglandins in transient inflammatory ocular hypertension**

Reiko Yamagishi-Kimura^1^, Megumi Honjo^1^, Makoto Aihara*^1^

^1^Department of Ophthalmology, The University of Tokyo School of Medicine,

Japan.

*Corresponding author:

Makoto Aihara, M.D., Ph.D.

Address:

Department of Ophthalmology,

The University of Tokyo School of Medicine,

7-3-1 Hongo Bunkyo-ku, Tokyo, 113-8655, Japan

Telephone number: +81-3-3815-5411

Fax number: +81-3-3817-0798

E-mail: [aihara-tky@umin.net](mailto:aihara-tky@umin.net)

Supplementary Table S1

The baseline IOP in WT, FPKO, EP1KO, EP2KO and EP3KO mice. Data are expressed as means ± SD (n = 9-10).

|  | **Wild type** | **FPKO** | **EP1KO** | **EP2KO** | **EP3KO** |
| --- | --- | --- | --- | --- | --- |
| Mean IOP±S.D. (mmHg) | 10.2±1.4 | 9.9±0.8  (N.S) | 10.6±0.9  (N.S) | 10.0±0.9  (N.S) | 9.4±0.9  (N.S) |

Supplementary Figure S1

The dose-dependence manner of PGE2 (A) and PGF2α (B) in WT mice. Data are expressed as means ± SD (n = 5). ** p < 0.01 for non-treated group versus each concentration of drugs treated group based on the Mann-Whitney U test. # p < 0.05 for 0.01% PGF2α versus 0.1% PGF2α based on the Student’s t-test.


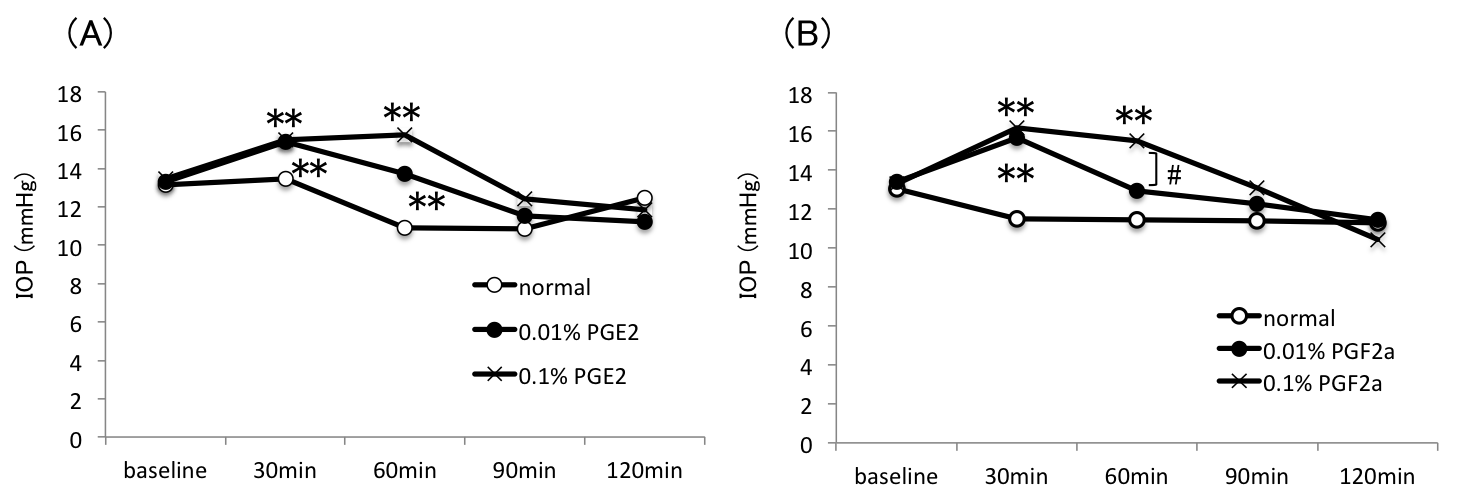

Supplement: Supplementary file 1 — Supplementary tableS1, Figure S1 [file 41598_2018_29273_MOESM1_ESM.docx]
